# DeviaTE: Assembly‐free analysis and visualization of mobile genetic element composition

**DOI:** 10.1111/1755-0998.13030

**Published:** 2019-07-03

**Authors:** Lukas Weilguny, Robert Kofler

**Affiliations:** ^1^ Institut für Populationsgenetik Vetmeduni Vienna Wien Austria

**Keywords:** assembly free, data visualization, divergence, mobile genetic element, python, transposon

## Abstract

Transposable elements (TEs) are selfish DNA sequences that multiply within host genomes. They are present in most species investigated so far at varying degrees of abundance and sequence diversity. The TE composition may not only vary between but also within species and could have important biological implications. Variation in prevalence among populations may for example indicate a recent TE invasion, whereas sequence variation could indicate the presence of hyperactive or inactive forms. Gaining unbiased estimates of TE composition is thus vital for understanding the evolutionary dynamics of transposons. To this end, we developed DeviaTE, a tool to analyse and visualize TE abundance using Illumina or Sanger sequencing reads. Our tool requires sequencing reads of one or more samples (tissue, individual or population) and consensus sequences of TEs. It generates a table and a visual representation of TE composition. This allows for an intuitive assessment of coverage, sequence divergence, segregating SNPs and indels, as well as the presence of internal and terminal deletions. By contrasting the coverage between TEs and single copy genes, DeviaTE derives unbiased estimates of TE abundance. We show that naive approaches, which do not consider regions spanned by internal deletions, may substantially underestimate TE abundance. Using published data we demonstrate that DeviaTE can be used to study the TE composition within samples, identify clinal variation in TEs, compare TE diversity among species, and monitor TE invasions. Finally we present careful validations with publicly available and simulated data. DeviaTE is implemented in Python and distributed under the GPLv3 (https://github.com/W-L/deviaTE).

## 1 INTRODUCTION

Transposable elements (TEs) are stretches of DNA that copy themselves within host genomes. They have been found in almost all eukaryotes and in most bacteria investigated so far (Biémont & Vieira, 2006; Wicker et al., 2007). TEs are important mutagens, which generate novel phenotypic variation; e.g., in *Drosophila melanogaster* an estimated 50%–80% of the observed mutations are due to TEs (Ashburner, Golic, & Hawley, 2005; Drake, Charlesworth, Charlesworth, & Crow, 1998). Transposons have been implicated in diverse phenomena such as human disease (Burns, 2017; Kazazian et al., 1988; Narita et al., 1993), environmental adaptation (Casacuberta & González, 2013; Schrader & Schmitz, 2019), genome evolution (Kazazian, 2004), quantitative variation (Mackay, Lyman, & Jackson, 1992) and domestication of important crops (Studer, Zhao, Ross‐Ibarra, & Doebley, 2011). Understanding TE biology is thus of vital interest for many different research fields.

Depending on the TE family and the host species, copy numbers can range from a few to hundreds of thousands of insertions (Biémont & Vieira, 2006; Pritham & Feschotte, 2007). Although defence mechanisms against these selfish elements have emerged (Brennecke et al., 2007; Yang, Wang, & Macfarlan, 2017), TEs have proven to be highly successful invaders. Hence, most genomes contain large fractions of TEs. In maize, for example, TEs account for a striking 85% of the genome (Schnable et al., 2009).

TE composition varies substantially among and within species (Bargues & Lerat, 2017; Bergman, Han, Nelson, Bondarenko, & Kozeretska, 2017), which could have important biological consequences. Variation in TE abundance among populations may be the hallmark of a recent TE invasion (Anxolabéhère, Kidwell, & Periquet, 1988; Kofler, Hill, Nolte, Betancourt, & Schlötterer, 2015) and may even drive speciation (Serrato‐Capuchina & Matute, 2018). Furthermore, some TEs exist as internally deleted variants, which act as suppressors of the full‐length TE (Black, Jackson, Kidwell, & Dover, 1987). The abundance of such internal deletions may vary among populations (Bergman et al., 2017), hence also the strength of TE repression may differ among populations. Variations of the sequence can highlight activity differences among samples, as base substitutions and indels within TEs could lead to elevated or reduced transposition rates (Beall, Mahoney, & Rio, 2002). Finally, terminally deleted insertions are likely immobilized (Marin et al., 2000), therefore variation in the prevalence of such terminal deletions may allow for identification of samples with inactive copies.

Despite this importance of TE variation, few tools exist that allow for the quantification of TE composition within and between species. Some tools for the analysis and visualization of TEs have been published, but most of them require a reference assembly and do not allow for a quantification of variation in sequence composition of the TE (Tempel & Talla, 2015; You et al., 2013). However, a high quality assembly is so far only available for a few species (e.g., 25 eukaryotic species; Lewin et al., 2018). Additionally, even if a reference assembly is available, resulting estimates of TE diversity may be biased because repetitive structures pose a significant challenge to assembly algorithms (Sohn & Nam, 2018), such that the variation and abundance of TEs will not be well captured in the resulting contigs. We therefore aimed to circumvent the need for a reference assembly and reasoned that aligning sequencing reads directly to consensus sequences of TEs will allow to infer accurate estimates of TE composition.

We implemented this approach in our novel program DeviaTE, a tool for an assembly‐free analysis of TE diversity. DeviaTE may be used to visualize and quantify TE abundance, single nucleotide poylmorphisms, indels and both internal and terminal deletions for multiple TE families and samples. It solely requires consensus sequences of TEs and sequencing reads (Sanger or Illumina) from one or more samples. DeviaTE may be used to study the TE composition of samples, assess TE divergence among species, monitor the progression of TE invasions and study clinal variation of TEs. Although DeviaTE was mainly designed for TEs, we note that it may also be used to analyse the composition of other genomic elements such as genes, gene families, viruses, bacteria and mtDNA.

## 2 MATERIALS AND METHODS

**Figure 1:**
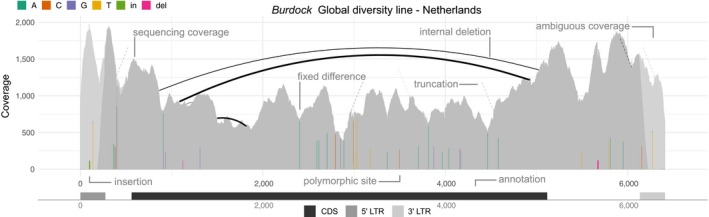
Example of the visualization of TE diversity with DeviaTE using burdock from *D. melanogaster*. Sequencing coverage is shown separately for unambiguously (dark grey) and ambiguously (light grey) mapped reads. Fixed differences and polymorphic sites are shown as coloured bars, with the height of the bar corresponding to the frequency of the SNP. The reference allele is not shown in the visualization. Internal deletions are displayed as arcs, where the width of the arcs scales with the abundance of the deletion. Terminal deletions are shown as dashed lines, with their opacity indicating the abundance of the deletion (darker lines indicate higher abundance). An annotation of the TE is shown at the bottom. Note that ambiguously mapped regions coincide with the long LTRs of burdock. Data are from a *D. melanogaster* line caught in the Netherlands (Grenier et al., 2015)

DeviaTE enables the analysis and visualization of the abundance as well as the genetic diversity of TE families. As input our tool requires consensus sequences of TE families and sequencing reads (Sanger or Illumina) from at least one sample, where samples could be individuals, pooled populations and tissues. DeviaTE provides quantitative estimates as well as a visual overview of TE diversity, which includes the coverage of ambiguously as well as unambiguously mapped reads, fixed and segregating polymorphisms (SNPs and indels) and internal and terminal deletions (Figure 1). Furthermore, the abundance of TEs is estimated if at least one single copy gene is included in the analysis.

An analysis of TE composition with DeviaTE proceeds in three steps (Figure S1). Reads first get quality‐filtered and aligned to a library of TE consensus sequences (FASTA format) using bwa‐sw (Li & Durbin, 2010). To obtain estimates of TE abundance, the sequence of one or more single copy genes may be added to the library of TE consensus sequences. Next, DeviaTE generates a table containing the abundance and diversity of TEs (coverage, SNPs, indels, internal and terminal deletions). Internally deleted TEs are inferred from subsequences of reads mapping to different reference positions (i.e., split‐reads). Initially, we evaluated the suitability of different mapping approaches to identify internal deletions. Interestingly, the local alignment algorithm bwa‐sw performed better than the two split‐read mappers, gsnap and minimap2 (Figure S2; Li, 2018; Li & Durbin, 2010; Wu & Nacu, 2010). Similarly to BLAST, bwa‐sw reports all possible local alignments, i.e., high‐scoring‐pairs (HSPs) of a read (Li & Durbin, 2010). These HSPs may be on different contigs, overlapping or separated by large gaps. To identify internal deletions it is thus necessary to arrange these HSPs into a single best contiguous alignment. Therefore, DeviaTE first constructs all possible combinations of HSPs and then removes combinations with overlapping subsequences and inconsistent alignments; e.g., when large internal regions of reads are not aligned (Figure S3). Finally, DeviaTE solely retains the combination of HSPs with the largest fraction of the read aligned and replaces all of the HSPs of a read by this best combination of HSPs. Since raw frequency estimates of internal deletions show a small read length dependent bias, DeviaTE automatically applies a correction factor (Figure 3d). The reason for this bias is that bwa‐sw does not align subsequences of reads that are shorter than 30 bp (by default). Hence, only internal deletions in central regions of reads can be detected.

To detect terminal deletions, DeviaTE utilizes soft clipped reads, i.e., reads for which a substantial fraction could not be mapped to any of the reference sequences. When the sequence of at least one single copy gene was provided, the tool also estimates the abundance of TEs by contrasting the total coverage between a TE and the single copy gene(s).

Notably, DeviaTE considers both the base and the physical coverage, i.e., the sequence spanned by split‐reads (Meyerson, Gabriel, & Getz, 2010). Such split‐reads may result from internally deleted TE insertions. This is important, as we found that a naive approach, which does not take the physical coverage into account, may lead to highly biased results (Figure S4).

Finally, the diversity of TEs is visualized with an illustration inspired by Sashimi plots, which are commonly used for quantitative visualization of splicing in RNA‐seq data (Katz et al., 2015). In our plots, internal deletions are shown instead of splicing events. The plots visualize the coverage of ambiguously and unambiguosly mapped reads, the frequency of SNPs, indels, internal deletions and terminal deletions (Figure 1). A panel showing features of the TE will be added at the bottom if a TE annotation is provided. In case several samples are analyzed, DeviaTE automatically arranges plots in a grid, in which different samples are aligned in rows and TE families in columns. To enable a comparison of TE abundance among samples the coverage may be normalized either to a million mapped reads or to the coverage of single copy genes. Normalization with the coverage of single copy genes may be especially useful when comparing TE abundance among species. Whenever the genome size varies among samples, normalization to 1 million mapped reads will result in misleading results, whereas normalization to single copy genes avoids this problem. The plots can be created in PDF or EPS format, which enables simple vector graphics processing.

DeviaTE is implemented in python (version 3.6+, Python Software Foundation, 2017) and distributed under the GNU GPLv3 License. It can be installed with the widely‐used pip python package manager. Additionally, a conda container‐type environment is available from the anaconda cloud. Conda sets up a separate environment and installs compatible versions of all dependencies of DeviaTE. Notably, the separate environment created by conda ensures that the installation does not interfere with other software and packages already present on the system. DeviaTE makes use of the python packages pandas version 0.23.4 (McKinney, 2010), pysam version 0.15 (Heger & Jacob, 2018) and samtools (Li et al., 2009). For visualization, DeviaTE uses r and the ggplot2 and cowplot packages (R Core Team, 2014; Wickham, 2016; Wilke, 2019).

## 3 RESULTS

**Figure 2:**
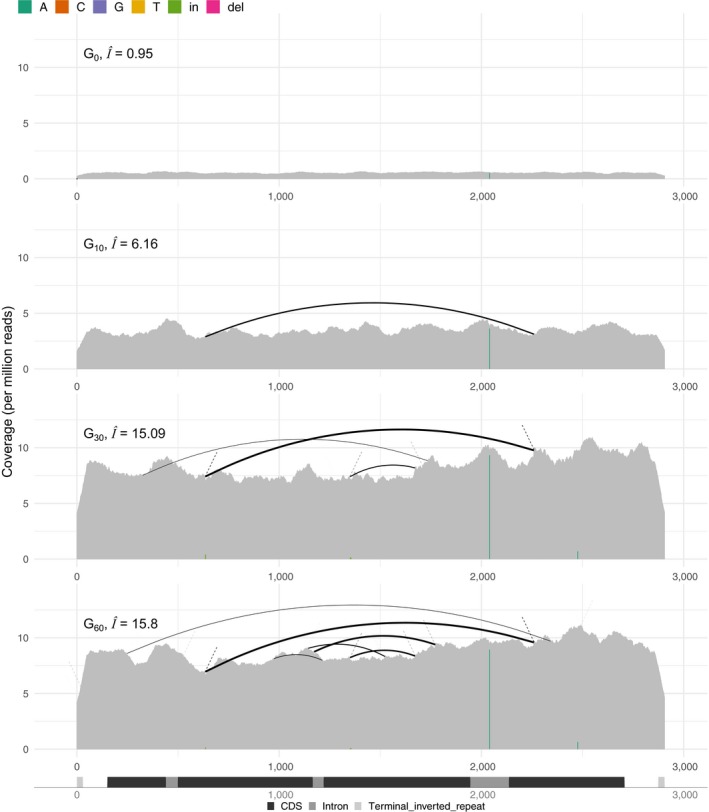
An invasion of the P‐element in an experimental *Drosophila simulans* population visualized with DeviaTE (data from Kofler et al., 2018). We show the abundance and the diversity of the P‐element for four successive time points. The coverage was normalized to one million mapped reads and estimates of insertions per haploid genome ($\hat{I}$) were calculated by relating the total coverage of the P‐element to the coverage of the gene *rpl32*. Note that the abundance of P‐elements as well as the number of internally deleted variants increases during the invasion

An analysis of TE abundance and diversity may be useful in many different research areas. DeviaTE may be used to study TE invasions (Figure 2), identify clinal variation in TE composition (Figure S6), estimate TE divergence within and among species (Figures S5, S7 and S8) and to estimate the proportions of internally deleted TEs. We demonstrate the utility of DeviaTE with a plot showing the composition of the long terminal repeat (LTR) retrotransposon burdock in a *D. melanogaster* population from the Netherlands (Figure 1: data from Grenier et al., 2015). This illustration visualizes the abundance as well as the diversity of burdock.

Further features of DeviaTE are demonstrated by using publicly available data of a P‐element invasion in experimentally evolving *D. simulans* populations (Figure 2: data from Kofler, Senti, Nolte, Tobler, & Schlötterer, 2018). The authors monitored a P‐element invasion for 60 generations by sequencing the populations every 10 generations as pools. To allow for a comparison of the TE abundance among samples we normalized the coverage to 1 million mapped reads.

DeviaTE automatically arranged the data from multiple generations into a vertical grid. A legend is shown at the top and the TE annotation at the bottom (Figure 2). Note the SNP at position 2040, which is characteristic for the *D. simulans* P‐element (Kofler et al., 2015; Yoshitake, Inomata, Sano, Kato, & Itoh, 2018). In agreement with Kofler et al. (2018), we observe an increase of P‐element copy numbers during the invasion as well as a rapid emergence of internally deleted P‐elements (Figure 2). Using the coverage of the single copy gene *rpl32* as reference, we estimate that the P‐element abundance increased from 0.95 insertions per haploid genome at the base population (G_0_) to 15.8 at generation 60 (G_60_, Figure 2). This is consistent with the estimates of Kofler et al. (2018), who relied on a different approach to estimate P‐element abundance, i.e., extrapolating the fraction of reads mapping to the P‐element to the estimated genome size of *D. simulans*.

DeviaTE also allows to normalize the coverage of TEs to the coverage of single copy genes. We demonstrate this feature by applying our tool to data from *D. melanogaster* populations sampled across the North American cline on the East Coast (Figure S6; data from Bergland, Behrman, O'Brien, Schmidt, & Petrov, 2014). We investigated whether copy numbers of the DNA transposon hobo exhibit clinal variation. Using the coverage of multiple single copy genes (*rpl32*,* piwi* and *act5C*) for normalization, we found a weak but non‐significant relationship between latitude and hobo copy numbers (Figure S6).

### 3.1 Validation

**Figure 3:**
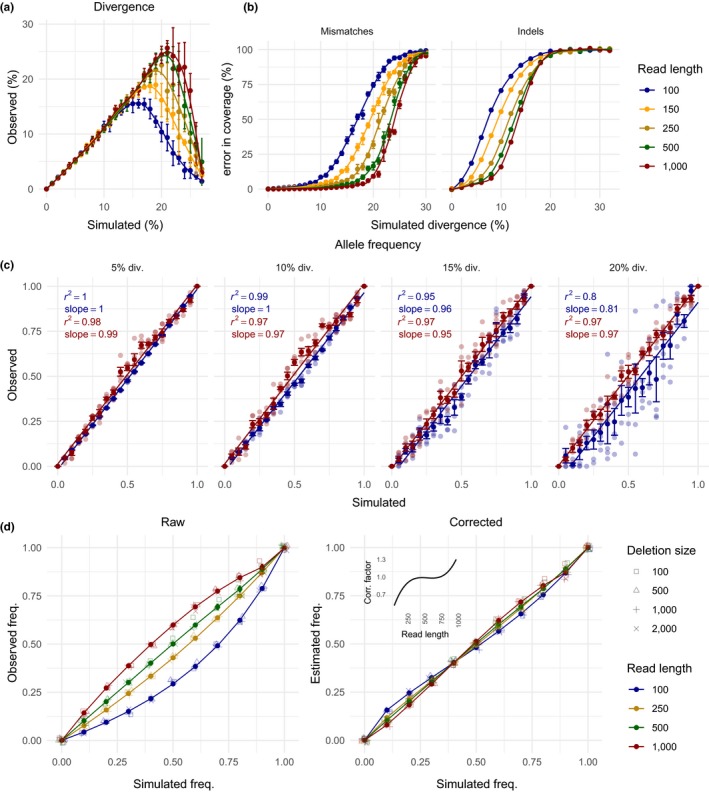
Validation of DeviaTE with simulated data. (a) Comparison between simulated and observed sequence divergence. DeviaTE accurately recovers simulated divergence of up to 15% for short reads (100 bp) and 22% for long reads (1,000 bp). Notably, the accuracy does not increase linearly with the read length. (b) Error of the estimated coverage dependent on the simulated divergence of reads. DeviaTE accurately reproduces the simulated coverage if the mismatch rate is smaller than 8% and 16% for short and long reads, respectively. Lower divergence levels are tolerated for indels. (c) Accuracy of allele frequency estimates dependent on the divergence. DeviaTE accurately reproduces allele frequencies of SNPs up to a divergence of 15%. (d) Accuracy of estimated frequencies of internal deletions. Since raw frequency estimates show a small bias (left), we implemented a read length dependent correction factor (right, inset), which substantially improves the accuracy of frequency estimates (right). Note that in a, c, and d a diagonal would indicate perfect agreement between expected and observed values

We carefully validated our tool with simulated data. First, we explored up to which level of sequence divergence DeviaTE accurately reports the expected TE diversity. We simulated transposable element landscapes with known levels of nucleotide and structural divergence using SimulaTE (Kofler, 2018). Briefly, we artificially inserted TEs into a nonrepetitive sequence, derived from chromosome 2R of *D. melanogaster*, and subsequently simulated sequencing reads of varying length from this template. We then tested the level of sequence divergence that is accurately reproduced by DeviaTE (Figure 3a). We found that our tool recovers divergence levels of up to 15% with short reads of 100 bp. An increase of the read length to 150 bp allows for the recovery of divergence levels up to 18%, whereas increasing read length further, results in less notable gains in accuracy (22% with 1,000 bp; Figure 3a). Next, we investigated the impact of diverged or erroneous sequences on the accuracy of the estimated coverage. We simulated TE insertions with known coverage and introduced various amounts of mismatches and indels into the reads. For short reads (100 bp), 10% mismatches led to a coverage error of 16%, whereas for long reads (1,000 bp) 10% mismatches resulted in a coverage error of merely 0.7% (Figure 3b, left). Less divergence is tolerated when indels are simulated instead of mismatches (Figure 3b, right).

To test the accuracy of the allele frequency estimates we simulated a population with 20 haploid genomes (Figure 3c). We used two TE sequences that differed solely by a single SNP and varied the frequency of these sequences in the population. At a moderate divergence (<10%) the allele frequency is reproduced faithfully (adj. *r* ^2^ = 0.99 for 100 bp reads, adj. *r* ^2^ = 0.97 for 1,000 bp reads), whereas for higher levels of divergence the accuracy diminishes (Figure 3c).

Next, we validated the frequency of internal deletions of TEs as estimated by DeviaTE. We simulated diverse internal deletions that varied in length, position within the TE, and population frequency. Raw frequency estimates show a read length dependent bias, which causes the frequency of internal deletions to be overestimated for long reads and underestimated for short reads (Figure 3d, left). To avoid these biases, DeviaTE automatically applies a correction factor that results in highly accurate frequency estimates (Figure 3d, right).

Finally we validated the performance of DeviaTE with publicly available data from the *D. melanogaster* strain ISO1. We annotated the assembly of this strain (r6.26) with RepeatMasker (Smit, Hubley, & Green, 1996‐2010) and estimated the TE abundance with DeviaTE using short read data (SRR8182349). The TE abundance estimated by DeviaTE correlates well with the number of insertions found by RepeatMasker and the number of insertions provided in the reference annotation (supplementary results; Figure S9).

### 3.2 Comparison to other programs

Several tools for analysing the TE composition of genomic data exist. They may broadly be classified into approaches that require a genome assembly (e.g., RepeatMasker [Smit et al., 1996‐2010], PoPoolationTE2 [Kofler, Gómez‐Sánchez, & Schlötterer, 2016]) and approaches that do not require an assembled genome. The latter category can be further divided into tools that perform a de novo assembly of reads (e.g., RepeatExplorer [Novák, Neumann, Pech, Steinhaisl, & Macas, 2013], dnaPipeTE [Goubert et al., 2015], RepLong [Guo et al., 2017]) and tools that align reads to TE sequences (e.g., DeviaTE; Table 1). These tools have different strengths and weaknesses. RepeatMasker and DeviaTE estimate the abundance of known TE families but may not identify novel TE families. On the other hand, de novo assembly based methods allow for the identification of novel families but the relationship between the resulting TE contigs and known TE families may be difficult to resolve (due to a complex network of one‐to‐many relationships between contigs and TE families). This may make it challenging to estimate the abundance of the known TE families. As an important advantage, tools that require a genome assembly usually allow to estimate the genomic position of TE insertions. However, a genome assembly is not available for many organisms and an assembly of low quality may lead to erroneous estimates of TE abundance and the genomic location of TEs (Table 1). To allow for a better overview of the strengths, weaknesses and applicability of different methods, we present a summary of the aforementioned programs in Table 1.

**Table 1:** Comparison of different tools for analyzing TE abundance. The required input, the resulting output, notable features and shortcomings are shown for each tool (RepeatMasker [Smit et al., 1996‐2010] RepeatExplorer [Novák et al., 2013], dnaPipeTE [Goubert et al., 2015], RepLong [Guo et al., 2017] and DeviaTE)

	DeviaTE	RepeatMasker	RepeatExplorer	dnaPipeTE	RepLong
Method	Alignment of reads to TEs	Alignment of TEs to assembly	De novo assembly	De novo assembly	De novo assembly
Input	Sequencing reads, TE sequences	Genome assembly, TE sequences	Sequencing reads, TE sequences	Sequencing reads (single‐end only), TE sequences, genome size estimate	Sequencing reads, genome size estimate
Output	Variation within TE families, visualization of TEs, quantification of variation, estimates of TE abundance	Annotation of repeats, masked query sequence, genome proportion of repeat orders, divergence to consensus	TE contigs, genome proportion of TEs, abundance of contigs	TE contigs, genome proportions of TEs, estimates of relative age of TEs, abundance of contigs	TE contigs
Notable features	Divergence at nucleotide resolution, short and long reads, detects structural variants of TEs, container‐type installation, read preprocessing	Identify low complexity DNA, detect contamination in assembly, different search engines	Platform independent Galaxy server, read preprocessing, protein domain search, identification of novel repeats, suitable for low‐coverage sequencing	Identification of novel repeats, suitable for low‐coverage sequencing	Supports long‐reads, sensitive algorithm, suitable for low‐coverage sequencing, no TE library required
Shortcomings	No genomic position of TEs, no novel repeats	No quantification of families, no novel repeats, susceptible to low assembly quality	No genomic position of TEs, long runtimes	Installation requires RepBase subscription, no genomic position of TEs, no direct quantification of families	No quantification of families, does not consider sequencing quality, no genomic position of TEs
Availability (Win/Mac/Linux)	−/+/+	−/+/+	+/+/+	−/−/+	−/−/+

## 4 DISCUSSION

Dependent on the length of the sequencing reads, DeviaTE allows to recover the abundance and diversity of TEs with divergence levels up to 22%. For very short reads (≈100 bp), the accuracy of DeviaTE suffers when the divergence of TEs exceeds 15% (Figure 3). This can potentially be an issue, as membership of TEs in a family depends on the sequence similarity. According to a TE classification proposed by Wicker et al. (2007), TEs belong to the same family if sequence similarity is at least 80%, over 80% of the sequence for at least 80 bp.

For evolutionary old TEs, an analysis with DeviaTE may thus be limited to recent and less diverged insertions. Hence, only a subset of ancient and highly degenerated TEs, such as L1 and CRE (Malik, Burke, & Eickbush, 1999), may be analysed. Old TEs are often found in pericentromeric and heterochromatin‐rich regions of the genome (Lerat, Rizzon, & Biémont, 2003). Thus, DeviaTE may show a reduced accuracy for TEs located in these regions. Another potential source for a bias might be TEs with structures prone to mutations. For example, Alu elements, which account for a staggering 11% of the human genome, contain an unstable A‐rich tail that rapidly shrinks during transposition and accumulates mutations (Deininger, 2011). However, some authors report the highest sequence divergence for purportedly highly‐divergent LINE and Alu element subfamilies to be 17.8% and 15.1%, respectively (Khan, Smit, & Boissinot, 2006; Price, Eskin, & Pevzner, 2004). These elements may be perfectly suited for an analysis with DeviaTE using reads of short or medium length.

As high quality genome assemblies are currently solely available for 25 eukaryotic species (Lewin et al., 2018), assembly‐free methods to quantify TEs, such as DeviaTE, may be useful for many different reasearch questions in model and non‐model organisms alike. DeviaTE, however, requires consensus sequences of TEs. Genomic reads are mapped to these consensus sequences and the TE abundance and diversity is estimated. The best results may thus be obtained with consensus sequences of high quality.

A comprehensive, high‐quality repository of repeat elements does not exist yet, but multiple efforts are pursued to achieve this goal. A widely‐used, standard database for sequences of repetitive elements is Repbase Update (Bao, Kojima, & Kohany, 2015). It contains the largest collection of consensus sequences for TEs and other repetitive elements, with currently over 44,000 entries from more than one hundred species. Other resources include Dfam with 4,150 entries (Hubley et al., 2016) and TREP, which initially contained TEs from *Triticeae* only, but was gradually extended with sequences from other plant and fungal species (Wicker, Matthews, & Keller, 2002; Wicker et al., 2007). Additionally, manually curated databases for diverse species or clades exist. These include collections for *Drosophila* (Bergman et al., 2018), conifers (Yi et al., 2018), fish (Shao, Wang, Xu, & Peng, 2018), and dioecious plants (Li et al., 2016). A comprehensive overview of available repositories is presented in Goerner‐Potvin and Bourque (2018).

However, if the sequence of a specific TE can not be found in any database, multiple tools for generating consensus sequences are available, e.g., RepARK, REPdenovo, RepeatScout, or RepLong (Chu, Pei, & Wu, 2018; Guo et al., 2017; Koch, Platzer, & Downie, 2014; Price, Jones, & Pevzner, 2005). These tools construct prototype sequences of repetitive elements from sequencing reads by assembling high‐frequency repeat k‐mers.

We hope that our novel tool DeviaTE will contribute to the investigation of TE dynamics in diverse species. Its strengths lie in the assembly‐free nature and wide applicability to sequencing reads of different technologies, lengths and from different sources, such as cells, tissues, individuals, and populations. DeviaTE is aimed to catalyze future progress in the broad spectrum of processes in which TEs play a major role.

## AUTHOR CONTRIBUTIONS

L.W. implemented and validated the software and wrote the manuscript. R.K. conceived the project and contributed to writing the manuscript.

## Data Availability

DeviaTE is open source and freely available at https://github.com/W-L/deviaTE. The tool may be installed using the pip or conda package managers. Installation instructions, a manual (https://github.com/W-L/deviaTE/blob/master/doc/MANUAL.md) as well as walkthroughs (https://github.com/W-L/deviaTE/blob/master/doc/WALKTHROUGH.md) are available. Code for the validation and benchmark can be found on our GitHub page.
