# Supplementary material for: DeviaTE: Assembly‐free analysis and visualization of mobile genetic element composition
Source: Mol Ecol Resour. 2019 Jul 3;19(5):1346–54. doi: 10.1111/1755-0998.13030 (PMC6791034; doi:10.1111/1755-0998.13030)
Supplement: Supplementary file 1 [file MEN-19-1346-s001.pdf]

MOLECULAR ECOLOGY
RESOURCES

Supplemental Information for:

DeviaTE: Assembly-free analysis and visualization of mobile genetic element composition

Lukas Weilguny[1] and Robert Kofler[1,*]
[1]Institut für Populationsgenetik, Vetmeduni Vienna, Wien, Austria
[*]Corresponding author: rokofler@gmail.com

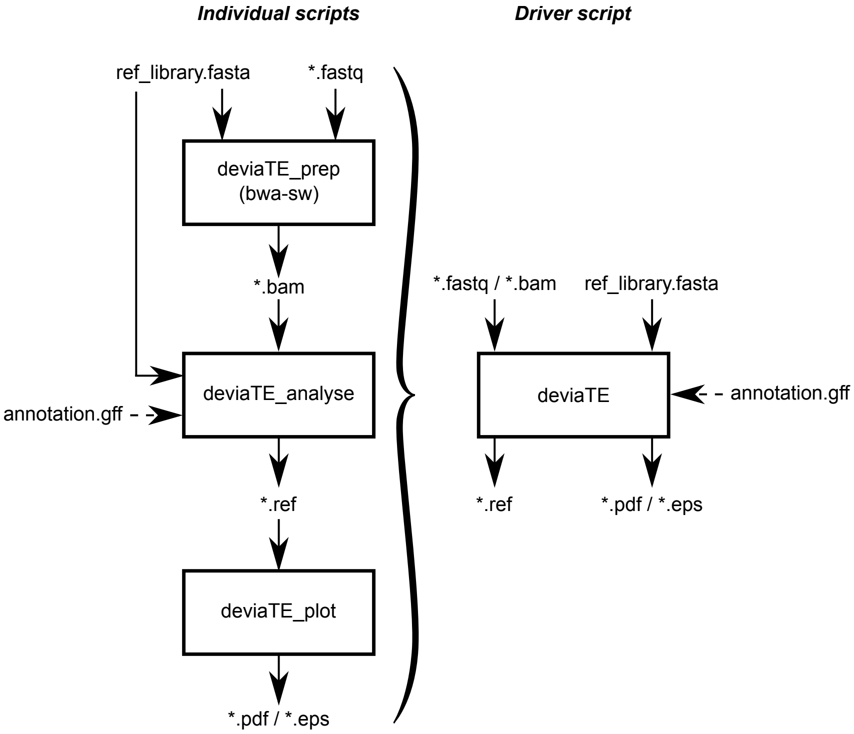

Figure S1: Workflow of DeviaTE. Mandatory input files are shown as solid lines and optional input as dashed lines. Boxes represent executable scripts. DeviaTE consists of three main scripts, to map the sequencing reads (deviaTE_prep), analyse a specific transposon family (deviaTE_analyse), and visualize the results (deviaTE_plot). This design allows for the analysis of several TE families with a single mapping step. A user-friendly driver script is provided that enables an analysis of multiple input files and transposon families. The input consists of sequencing reads (*.fastq), a library of reference sequences (ref_library.fasta) and an optional annotation of the reference sequences. As output DeviaTE provides a table containing information on the analysed TE families (*.ref) and a visualization (*.pdf/*.eps).

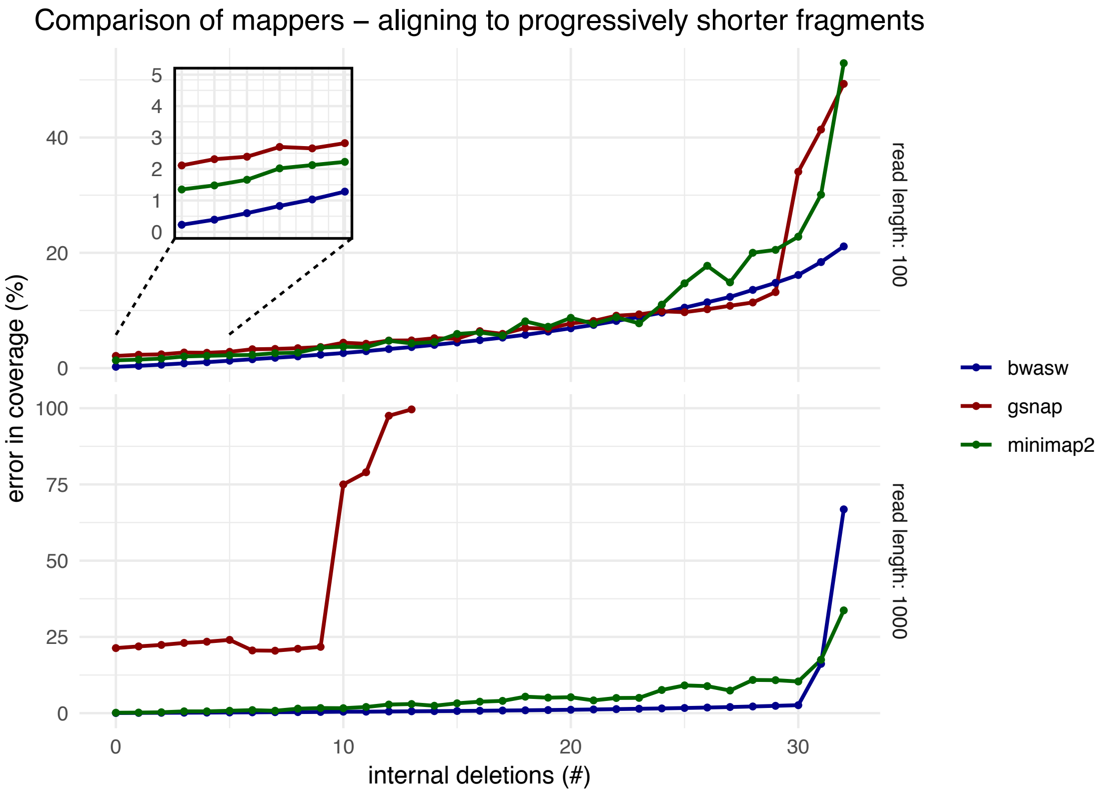

Figure S2: Suitability of different mapping algorithms for aligning reads to internally deleted TEs. The assembly-free nature of our approach necessitates reliable alignments to short reference sequences. We therefore compared three widely-used mapping tools and measured the error in coverage. We introduced a variable amount of internal deletions into TE sequences from the Porto1 family, generated reads for these sequences and aligned them back to the consensus sequence of the TE. We found that bwa-sw reproduced the simulated coverage more accurately than gsnap and minimap2 (Li & Durbin, 2010; Wu & Nacu, 2010; Li, 2018).

Algorithm 1 Find internal deletions from split reads

```
for read in alignment do
    segments ← all mappings of read
    if segments > 1 then
        macs ← Powerset(segments)
        for m in macs do
            overlap ← CheckOverlap(m)
            distance ← CheckDistance(m)
            if overlap ≥ limit then
                Discard(m)
            else if distance ≥ limit then
                Discard(m)
            end if
            scores ← CumulativeQuality(m)
        end for
        HighScoringMac ← max(scores)
        NewMapping ← BuildRead(HighScoringMac)
    end if
end for
```

Figure S3: Algorithm to detect internal deletions. To find the best contiguous alignment, all subsequences of a single read mapping to a reference sequence are used to construct all possible combinations of the aligned subsequences, here called multiple alignment candidates (macs). After checking for overlaps and inconsistent gaps within reads, the algorithm searches for the macs with the largest fraction of aligned bases. Notably, BuildRead generates a novel sam entry based on the highest scoring mac, which replaces all previous subsequences. This step also constructs a new CIGAR string from the subalignments.

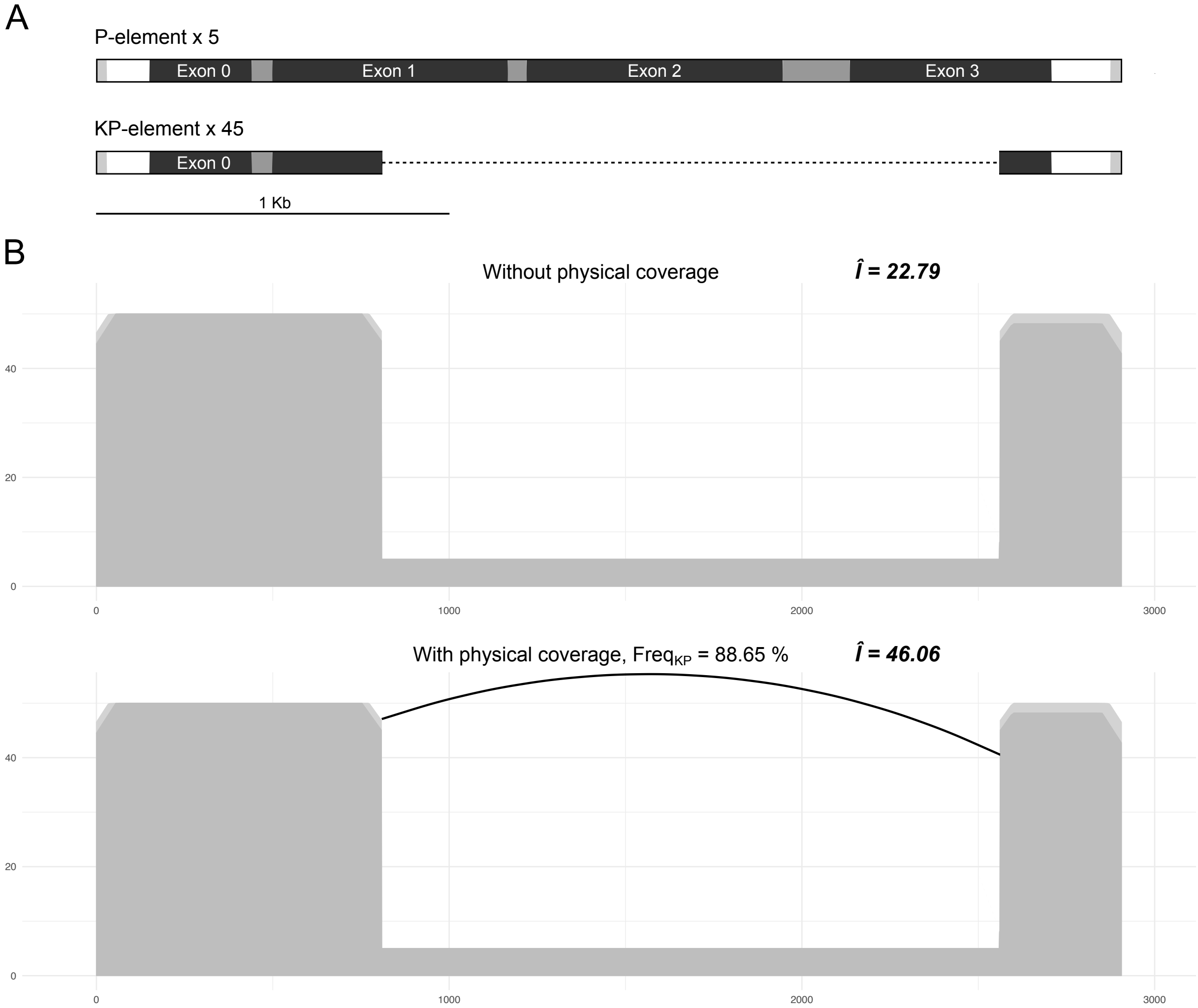

Figure S4: Ignoring the physical coverage of TEs leads to biased estimates of TE insertion numbers. A) Structure of the full-length P-element and the internally deleted KP element. We simulated reads from a single *Drosophila melanogaster* chromosome containing fifty insertions of the P-element, five of which were full-length and the remaining 45 were internally deleted KP elements. B) Insertion copy numbers were estimated with DeviaTE using the single copy genes *rpl32, piwi* and *act5C* for normalization. A naive approach that ignores the physical coverage finds a mere 22.79 insertions. However, with our approach including physical coverage, DeviaTE identifies 46.06 P-element insertions (out of 50).

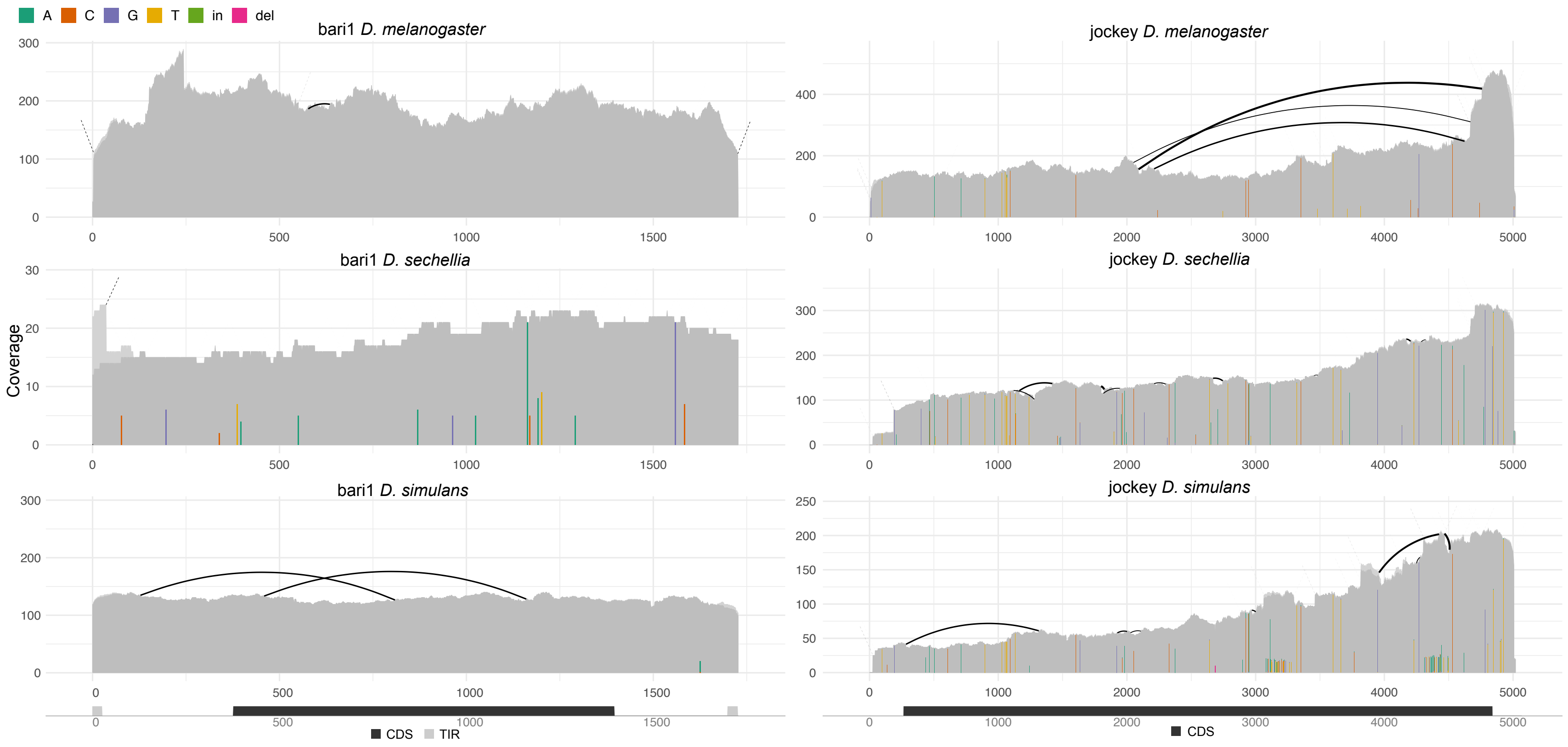

Figure S5: DeviaTE allows to compare the TE composition among species. As examples we show plots for bari1 and jockey in the genomes of *Drosophila melanogaster, D. sechellia* and *D. simulans* (data from Drosophila 12 Genomes Consortium et al., 2007). Bari1 (left column) is a 1.7 kb terminal inverted repeat element that is widespread in the *Drosophila* genus. The copies show high sequence homology across species with few low frequency polymorphisms and internal deletions. The transposon jockey (right column) is a LINE-like non-LTR retrotransposon of 5 kb length. It has a higher level of divergence among species as we find fixed differences and large deletions in all three species. Some fixed differences are shared, while others are exclusive to one species. Interestingly, the skewed distribution of the sequencing coverage was described before (Kaminker et al., 2002) and may be explained by interrupted replication resulting from the transposition mechanism used by LINE-like elements (Finnegan, 1997). The notable difference in sequence divergence between these TE families may be indicative of the age of the families. Whereas horizontal transfer is frequent in DNA transposons such as bari1, non-LTR transposons such as jockey are thought to evolve solely vertically and thus show high levels of sequence divergence (Bartolomé et al., 2009; Malik et al., 1999).

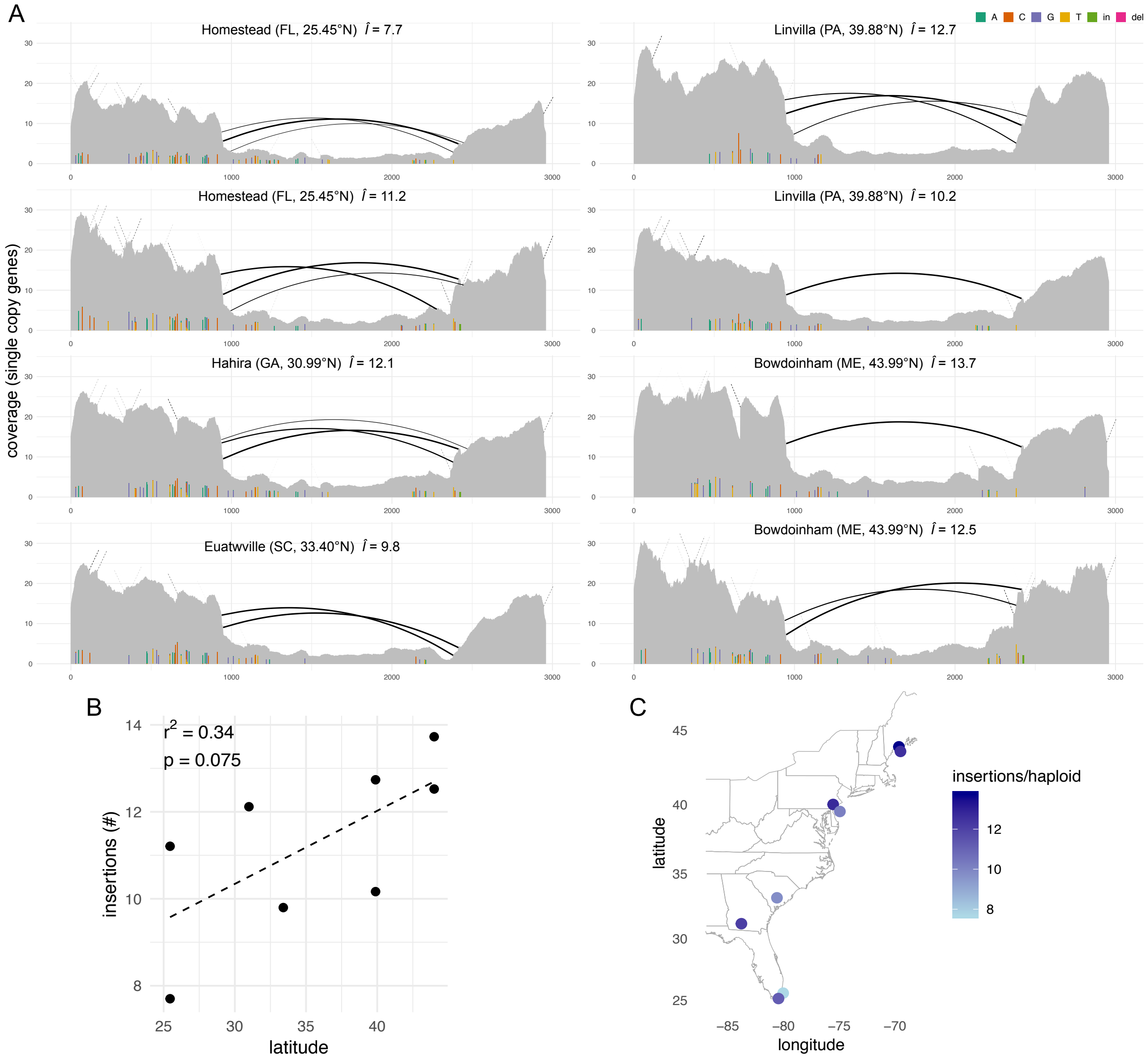

Figure S6: DeviaTE may be used to study clinal variation in TE composition. In this example we test whether the transposon hobo shows clinal variation in *Drosophila melanogaster* populations sampled along the North American East Coast (data from Bergland et al., 2014). A) Diversity and estimated insertion numbers per haploid genome ($\hat{I}$) of the hobo element in eight populations ranging from Florida (southern-most samples, top left) to Maine (northern-most samples, bottom right). Estimates are obtained by relating the coverage of the single copy genes *rpl32, piwi* and *act5C* to the TE coverage. The lowest number of insertions was estimated for a sample from Homestead (FL) at 7.7 copies, whereas the highest number of insertions, 13.7, was found in a sample from Bowdoinham (ME). B and C) A weak but non-significant relationship between hobo abundance and latitude was found.

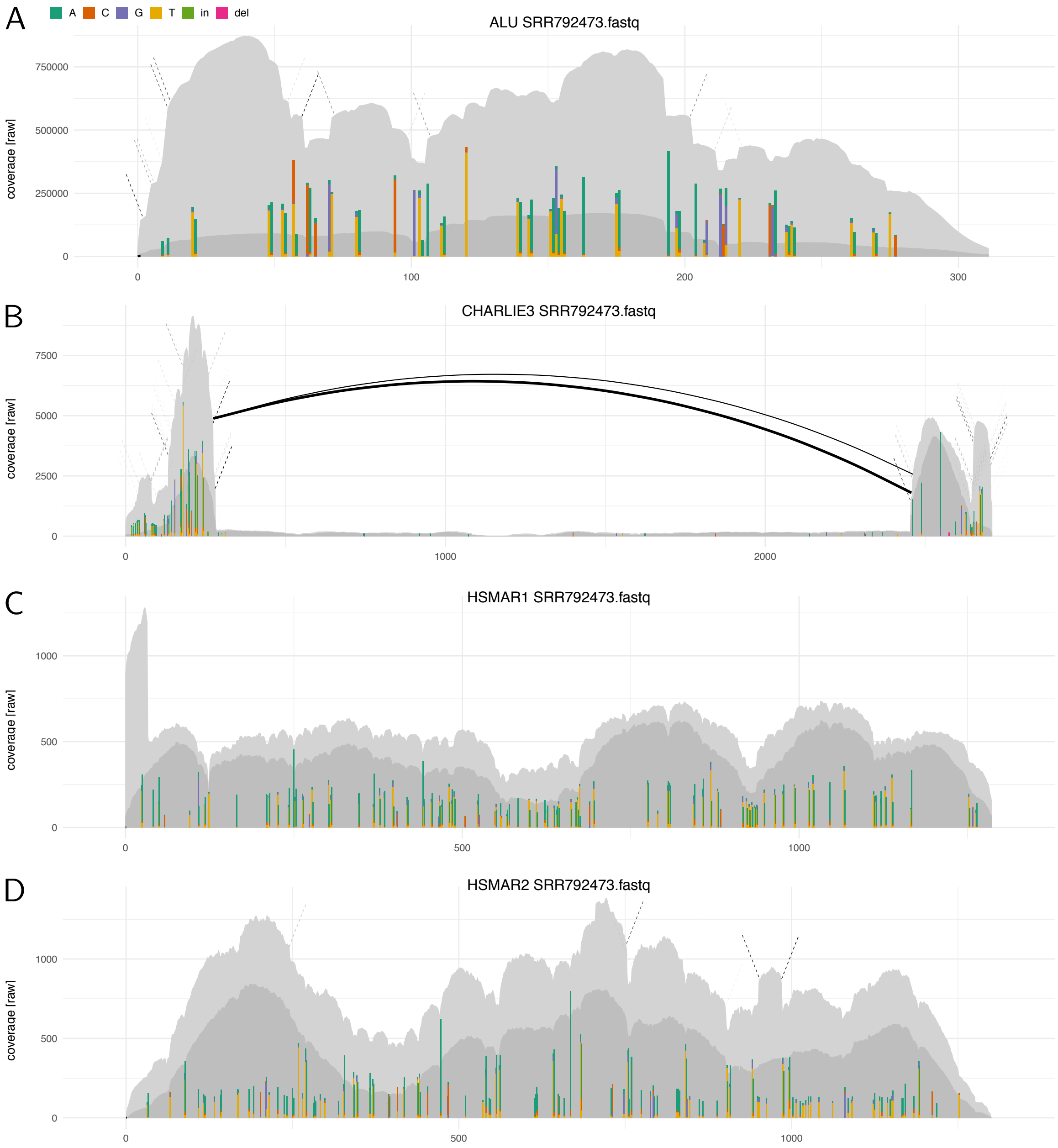

Figure S7: Four transposable elements of humans visualized with DeviaTE A) The Alu element is a primate specific, non-autonomous short interspersed element (SINE). With over one million copies, it is one of the most prevalent and successful TEs (Deininger, 2011). Their evolutionary old age and instable A-tail lead to highly ambiguous coverage. B) Charlie3 is a DNA transposon, which belongs to the hAT superfamily. Similar to other DNA transposons, we observe a high proportion of internally deleted variants and only few full-length copies. C and D) HSMAR1 and HSMAR2 are ancient, human-specific mariner elements. Our results suggest that these TEs have few internal deletions. Data are from Illumina whole genome sequencing of a Finnish individual in the 1000 Genomes Project (SRR792473, The 1000 Genomes Project Consortium et al., 2015) Consensus sequences were obtained from Repbase (Bao et al., 2015).

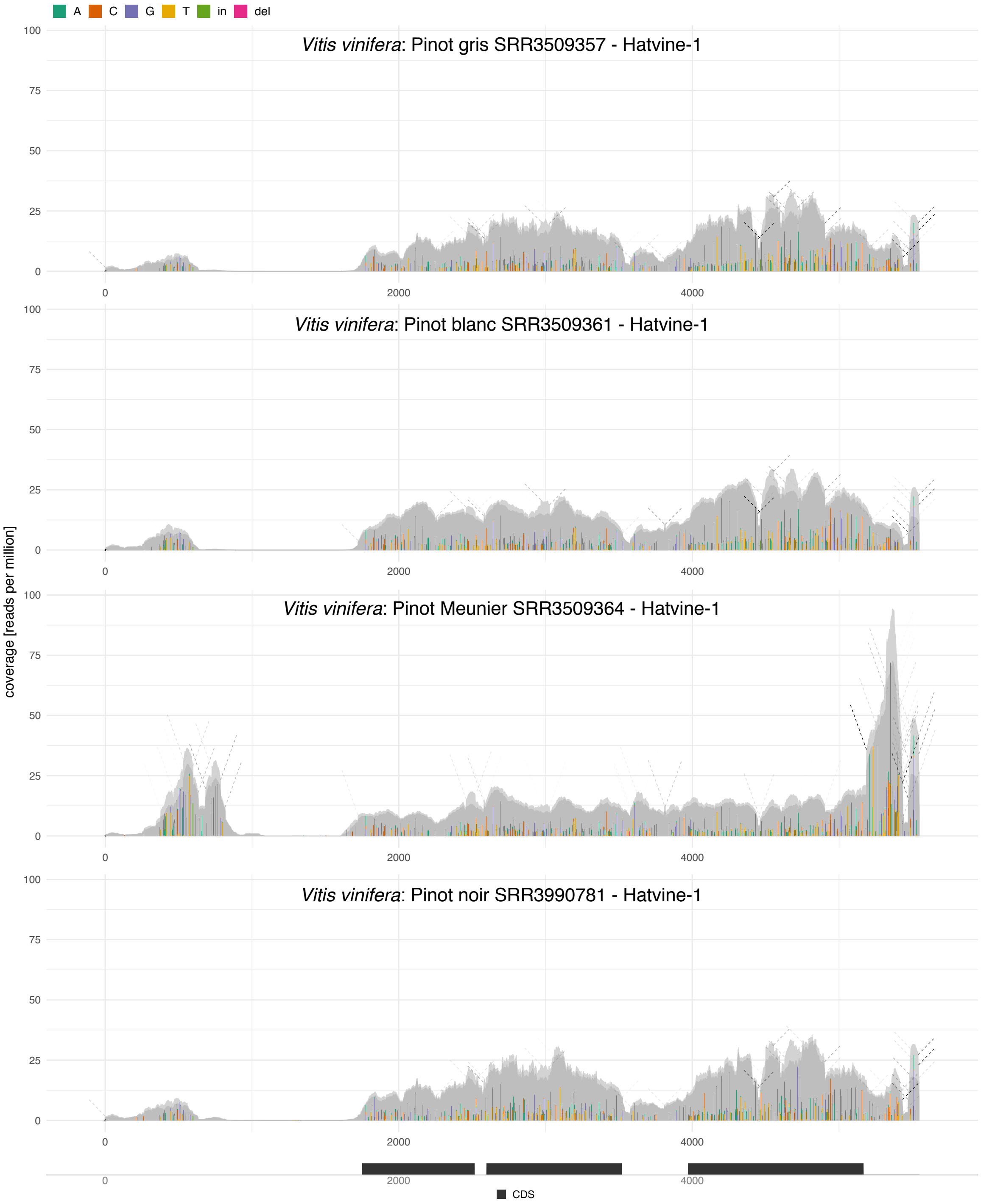

Figure S8: Comparative analysis of Hatvine-1 in four varieties of *Vitis vinifera*. We selected four commercially significant cultivars of the common grapevine in a dataset from Marroni et al. (2017). Transposons are thought to play a major role in the evolution and domestication of grapevine and possibly contribute to shaping the different varieties (Benjak et al., 2008). Hatvine-1 belongs to the ancient hAT superfamily, which encompasses the most prevalent TEs in the genome of *V. vinifera*. Interestingly, our results show an increased coverage of the coding regions, but we do not find conclusive evidence for complete elements, as suggested by Benjak et al. (2008). The consensus sequence for Hatvine-1 was obtained from Repbase (Bao et al., 2015).

Supplementary Results: benchmark

To benchmark the performance of DeviaTE with real data, we compared the number of insertions found with RepeatMasker to estimates obtained with DeviaTE. RepeatMasker (version 4.0.8, RMblast search engine) was used to quantify TE insertions in the reference genome of *Drosophila melanogaster* (ISO1 strain; Flybase; r6.26; Thurmond et al., 2018). We used the `-nolow` parameter to ignore low-complexity DNA and utilized TE consensus sequences from Bergman et al. (2018). For the analysis with DeviaTE, we used whole-genome sequencing reads from *Drosophila melanogaster* strain ISO1 (SRR8182349) and the same library of TE reference sequences. We used the single-copy gene normalization strategy of DeviaTE and provided the following single-copy genes: act5C, rpl32, RpII140, piwi and p53. In the raw output of RepeatMasker, the identified TE regions may be overlapping and highly fragmented, even for sequences that are likely derived from the same TE insertion. Additionally, RepeatMasker reports fragments of rather short length (< 25 aligned nucleotides) and high divergence (> 35 %). This may lead to a severe overestimation of the insertion numbers in the raw output. To demonstrate this, we included single-copy genes (act5C, rpl32, RpII140, piwi and p53) into the repeat library of RepeatMasker. The fragment counts reported by RepeatMasker for these single-copy genes ranged from 2 to a staggering 1427. To resolve these problems, we merged overlapping matches and filtered short and highly diverged matches (code available on GitHub). We used the single-copy genes to calibrate the parameters of the filtering step, such that solely the correct copy of the genes was retained (correct position and gene). Finally, we applied this filtering procedure to the TE insertions, using the parameters found for the single copy genes. The insertion numbers of the reference annotation were obtained from Flybase (r6.26; Thurmond et al., 2018). Data were log10 transformed and correlation was assessed by non-parametric Spearman's ranked correlation. We found that the estimated insertion numbers of DeviaTE correspond well to the filtered output of RepeatMasker and the TE annotation in Flybase, but only moderately to the raw values reported by RepeatMasker (Fig. S9).

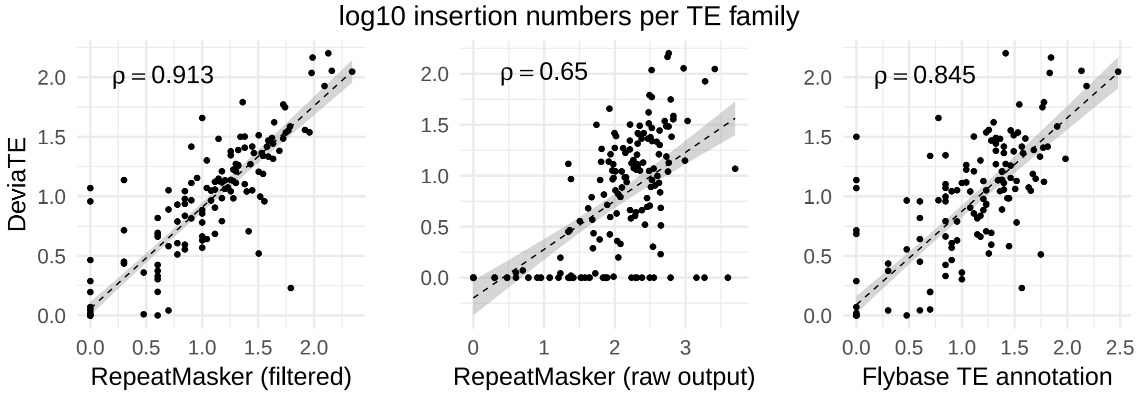

Figure S9: Comparison of TE copy numbers in *D. melanogaster* found by DeviaTE and RepeatMasker. We quantified insertion numbers for each TE family (dots). Additionally, we used the reference annotation of TEs in *D. melanogaster*. The results of DeviaTE correlate well with the filtered RepeatMasker output and the reference annotation, but only moderately with the raw output of RepeatMasker (correlations are Spearman's rho). Filtering was necessary as RepeatMasker reports overlapping and highly fragmented matches. See supplementary note for details on the filtering strategy.

References

Bao, W., Kojima, K. K., & Kohany, O. (2015). Repbase Update, a database of repetitive elements in eukaryotic genomes. *Mobile DNA*, *60*(1), 11.

Bartolomé, C., Bello, X., & Maside, X. (2009). Widespread evidence for horizontal transfer of transposable elements across *Drosophila* genomes. *Genome Biology*, *10*(2), R22.

Benjak, A., Forneck, A., & Casacuberta, J. M. (2008). Genome-wide analysis of the "cut-and-paste" transposons of grapevine. *PLOS ONE*, *3*(9), 1-14.

Bergland, A. O., Behrman, E. L., O'Brien, K. R., Schmidt, P. S., & Petrov, D. A. (2014). Genomic evidence of rapid and stable adaptive oscillations over seasonal time scales in Drosophila. *PLOS Genetics*, *10*(11), e1004775.

Bergman, C. M., S., H., Benos, T., Bayraktaroglu, L., Ashburner, M., de Grey, A., ... Kaminker, J. (2018). Drosophila transposable element consensus sequences - v10.1. *https://github.com/cbergman/-transposons*.

Deininger, P. (2011). Alu elements: know the SINEs. *Genome Biology*, *12*(12), 236.

Drosophila 12 Genomes Consortium, Clark, A. G., Eisen, M. B., Smith, D. R., Bergman, C. M., Oliver, B., ... MacCallum, I. (2007). Evolution of genes and genomes on the Drosophila phylogeny. *Nature*, *450*, 203-218.

Finnegan, D. (1997). Transposable elements: How non-LTR retrotransposons do it. *Current Biology*, *7*(4), R245–R248.

Kaminker, J. S., Bergman, C. M., Kronmiller, B., Carlson, J., Svirskas, R., Patel, S., ... Celniker, S. E. (2002). The transposable elements of the *Drosophila melanogaster* euchromatin: a genomics perspective. *Genome Biology*, *3*(12), RESEARCH0084.

Li, H. (2018). Minimap2: pairwise alignment for nucleotide sequences. *Bioinformatics*, *34*(18), 3094–3100.

Li, H., & Durbin, R. (2010). Fast and accurate long-read alignment with Burrows–Wheeler transform. *Bioinformatics*, *26*(5), 589–595.

Malik, H. S., Burke, W. D., & Eickbush, T. H. (1999). The Age and Evolution of Non-LTR retrotransposable elements. *Molecular Biology and Evolution*, *16*(6), 793-805.

Marroni, F., Scaglione, D., Pinosio, S., Policriti, A., Miculan, M., Di Gaspero, G., & Morgante, M. (2017). Reduction of heterozygosity (ROH) as a method to detect mosaic structural variation. *Plant Biotechnology Journal*, *15*(7), 791-793.

The 1000 Genomes Project Consortium, Campbell, C. L., Scheller, C., Horn, H., Kidd, J. M., Doddapaneni, H., ... Fitzgerald, T. W. (2015). A global reference for human genetic variation. *Nature*, *526*(7571), 68–74.

Thurmond, J., Goodman, J. L., Strelets, V. B., Attrill, H., Gramates, L. S., Marygold, S. J., ... FlyBaseConsortium (2018). FlyBase 2.0: the next generation. *Nucleic Acids Research*, *47*(D1), D759-D765.

Wu, T. D., & Nacu, S. (2010). Fast and SNP-tolerant detection of complex variants and splicing in short reads. *Bioinformatics*, *26*(7), 873–881.
